# Co‐Producing the Australian Functional Strengths and Support Needs Framework: Approach, Experience and Outcomes

**DOI:** 10.1111/hex.70791

**Published:** 2026-07-31

**Authors:** Emmah Baque, Rachelle Wicks, Zheng Yen Ng, Libby Groves, Bianca Comfort, Amanda Curran, Clare Gibellini, Ailsa Leslie, Erin West, Kiah Evans, David Trembath

**Affiliations:** ^1^ School of Allied Health, Sport and Social Work Griffith University Gold Coast Australia; ^2^ CliniKids, The Kids Research Institute Australia Perth Australia; ^3^ Australian EDS & HSD Network Melbourne Australia; ^4^ Comfort Psychology Melbourne Australia; ^5^ Australian Association of Psychologists Inc North Melbourne Australia; ^6^ People with Disability Australia Sydney Australia; ^7^ Occupational Therapy Melbourne Australia; ^8^ Speech Pathology Australia Melbourne Australia; ^9^ School of Health and Clinical Sciences University of Western Australia Perth Australia

**Keywords:** approach, co‐production, evaluation, experience, outcomes, participatory research

## Abstract

**Background:**

Participatory research, including co‐production, is highly valued in health‐ and community‐focused research. Evaluating outcomes of co‐production activities is essential to informing and improving future efforts.

**Objective:**

The study aimed to evaluate the experiences of participants involved in co‐production to develop a National Framework for assessing children's functional strengths and support needs. The National Framework was developed to guide professionals when working with children aged 0–12 years, including children who do and do not have one or more diagnosed condition(s), and their families in Australia and inform good policy across health, education, disability, and community support sectors.

**Design:**

We used a cross‐sectional descriptive survey study design.

**Setting and Participants:**

We invited representatives of 23 community and professional organisations who were involved in co‐production, to complete an online survey. Thirteen individuals accepted the invitation.

**Main Variables Studied:**

Participants provided quantitative and qualitative reflections regarding the extent to which the co‐production process was *accessible, inclusive, respectful, participatory, helpful* and *outcomes focussed*.

**Main Outcome Measures:**

Data from the online survey were analysed using descriptive statistics and open‐ended responses used to illustrate the findings.

**Results:**

Participants reported the co‐production process was highly positive, inclusive and respectful. Most reflected positively on their experience (e.g., accessibility, inclusiveness) and outcomes (e.g., open to refining ideas, aims achieved). However, some variability was noted due to the consensus‐seeking approach used which resulted in not all individual contributions being fully reflected in the final product.

**Discussion:**

The findings provide further evidence of the value and importance of co‐production for designing and delivering community‐focused initiatives. Recommendations for further enhancing the approach, experience, and outcomes of co‐production activities for the individuals involved are provided. These include establishing detailed documentation of the co‐production methods used, establishing participants' expectations, and supporting open discussion of what went well/not well in co‐production.

**Conclusion:**

This study shows participant perspectives on the co‐production process and the importance of considering co‐production outputs, experiences, and outcomes to understand such perspectives. Examples of strategies that can support well‐organised, respectful and purposeful engagement are provided.

**Patient or Public Contribution:**

The participants include people with lived experience advocating for individuals and professionals. Three authors report first‐hand lived experience of disability.

## 1 Introduction

Participatory research is highly regarded in health, disability, education, and community services research because of the range of benefits for individuals involved and the positive outcomes [1, 2, 3, 4]. Participatory research methods focus on consumer identified priorities and solutions, thus maximising the likelihood of appropriate, acceptable, and sustainable outcomes [5, 6]. Consumer involvement can take many forms, including co‐creation, co‐design, and co‐production. While methods of consumer and community involvement in participatory research differ in practical ways ‐ such as at which points collaboration occurs and in what form – they all have in common the commitment to partner with key stakeholders in the research and development process [1, 7]. However, genuine participatory research must be principles‐based and authentic [8]. It takes time, including to build the research skills required, as well as the relationships and trust that are the foundation for appropriate, effective, and respectful collaboration [3, 7]. Importantly, participatory research also involves commitment to ongoing evaluation and improvement of the methods used, to ensure they are fit for purpose for the communities and contexts in which they are applied.

Fortunately, there is a substantial body of research that can inform the design and implementation of participatory research methods. For example, Grindell, Coates [2] completed a systematic review of co‐production, co‐design, and co‐creation methods used in the management of health conditions. Based on findings from 24 studies, they identified key aspects (e.g., bringing people together as active and equal partners), proposed mechanisms of action (e.g., giving everyone a voice and sense of ownership), and activities (e.g., interviews and creative workshops). Duea, Zimmerman [9] provide a guide for selecting participatory research methods in the field of health research that considers the project and partnership goals. The authors categorised methods into five domains: (1) *engagement and capacity,* (2) *building*, (3) *exploration and visioning*, (4) *visual and narrative, mobilisation*, and (5) *evaluation*. The *evaluation* domain is particularly relevant for the current study to assess the approach, experiences of participation, and project outcomes. As Duea, Zimmerman et al. highlight, evaluating outcomes of participatory research methods is important and are ideally suited to evaluating the outcomes of participatory research projects.

Despite the importance of evaluating participatory research methods, there has been a lack of consistency in the outcome measures used. This was a key finding reported by Nordin, Kjellstrom [10], following their systematic review of measurement and outcomes of co‐production in health and social care. The authors examined the methods used across 43 studies conducted in 12 countries. They noted that co‐production was associated with positive experiences and learning but also highlighted a distinct lack of consistency in measurement itself. This finding likely reflects the variety of applications of participatory research [9], but also the inherent value these methods place on adopting approaches (including outcome measurement) relevant to the specific context and community in which they are used. Nordin, Kjellstrom [10] proposed a solution to the challenges associated with outcome measurement. They suggested evaluation should focus on three perspectives related to co‐production: *outputs* (what is produced), the *experiences* of those who participate, and *outcomes* (the impacts of what is produced). Such an approach can accommodate both established and study‐specific measures, within a framework that supports the synthesis of findings across studies to inform further improvements over time [10].

In line with Nordin, Kjellstrom's [10] recommendations, this study sought to address the aforementioned challenges in outcome measurement by evaluating the co‐production methods used within the development of the *National Framework for assessing children's functional strengths and supports needs in Australia* (herein referred to as the National Framework) in terms of both Reference Group member experiences, and the outputs and outcomes of the co‐production process. The National Framework aims to improve children's health, activities and participation by setting out an evidenced based, culturally appropriate approach for assessment, differentiation and written reporting of children's functional strengths and support needs. It was developed to guide professionals when working with children aged 0–12 years, including children who do and do not have one or more diagnosed condition(s), and their families in Australia, and inform good policy across health, education, disability, and community support sectors. Conducted predominantly online, the participatory research process involved representatives from 23 community and professional organisations. Our specific aim of the co‐production evaluation study was to examine participants' reflections on the approach, their experiences, and project outcomes to inform future co‐production activities.

## 2 Method

### 2.1 Design

We used a cross‐sectional descriptive survey study design to explore participants' reflections on their involvement in a participatory research process. The study received ethical approval from The Griffith University Human Research Ethics Committee (GU Ref No: 2024/814) which included coverage for research participants (i.e., members of the co‐production group who volunteered to engage in this follow‐up study) to both identify themselves and act as co‐authors.

### 2.2 Context

As noted, this study was conducted within a broader programme of research to develop the National Framework [11]. The programme of work occurred over a 12‐month period, and included a systematic review of research literature, international environmental scan of tools and frameworks, community consultation via an online survey, yarning sessions via Microsoft Teams, and co‐production with representatives of the 23 community and professional organisations. Individual representatives brought to the group a range of personal and professional experiences, including as persons with disability, family members and associates of persons with disability, and professional experience across health, education, disability, and community sectors. In the context of this work, co‐production was defined as *‘…researchers and end‐users working together as peers to ensure the purpose of the research, the research methodology and the application of research outputs are relevant to, and appropriate for the end users’* [12].

**Figure 1:**
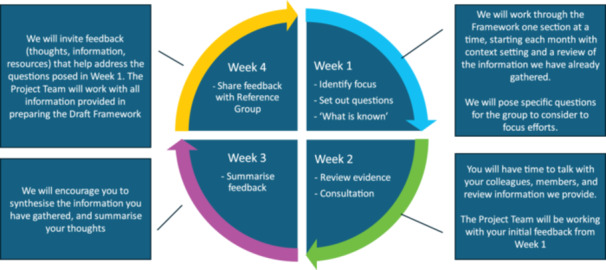
The co‐production cycle.

A detailed description of the co‐production process is contained in the Supporting Information document [13] that accompanies the National Framework, including people involved, governance, terms of reference, processes, activities, information gathered, and findings. However, for context and in brief, co‐production occurred via 14 online, regular scheduled meetings between February to September 2024, as well as via direct consultation, asynchronous information gathering, and document review activities. Central to this process was the use of *co‐production points* (CPPs) which provided a structure for identifying an *issue of interest*, what was *already known* (e.g., research evidence, environment scan), *what was needed* (e.g., ideas, reflections, information from co‐production members), *what activities* would occur (e.g., small group discussion at an online meeting), and following the activities *what was found*. As illustrated in Figure 1, each CCP was worked through over a 4‐week period, with the opportunity for further iterations if required. An example of a CCP is provided in Appendix 1.

The 14 online meetings followed a consistent structure: (a) meeting introduction (acknowledgement of Country, meeting overview, minutes, updates); (b) review of previous CPPs in meetings; (c) CPP presentations (e.g. findings from environmental scan, drafts of Framework and associated documents); (d) CPP small break‐out group discussions; (e) CPP whole‐group discussions; (f) CPP polls and/or surveys; (g) final comments in meetings; and (h) review of draft documents. The process generated 31 h, 38 min of recordings (17;10 for main room, 14;28 for breakout rooms) which were transcribed and analysed, and over 175 pages of summarised evidence (see Supporting Information; 13). Information gathered from CPPs and other organisation input provided to the research team across time were collated with results from the systematic and grey literature review, online community consultation, the broader literature and discussions with the research team to develop each section of the Framework (see Supporting Information; 13). For example, quotes from meeting transcripts were used verbatim when developing text for the Guiding Principles and thus reflected the input of Reference Group member contributions directly.

Reference Group members reviewed two draft versions of the Framework document as part of co‐production activities. For Draft 1, a feedback template and evaluation scale were used per section of the Framework to gather feedback on any issues identified, proposed solutions, and the degree of agreement (*strongly disagree, disagree, neutral, agree, strongly agree*) that information in each section was *accurate, complete, respectful, helpful*, and *likely to be consistent with their organisation's and/or communities' views, preferences, and priorities*. Members provided their responses via a shared Word document, an online survey, and/or individual conversations with members of the research team. Feedback was collated and systematically addressed, and amendments made to the draft document accordingly. All feedback that fell within the scope of the resource aim was implemented. To maintain accountability and transparency, reasons for not adopting specific feedback were shared with relevant members. A summary of feedback and edits made were also shared with the broader group and provided opportunity for further feedback and/or discussion during meeting 13. This process was intended to assist in contextualising the edits and make the process of reviewing Draft 2 more straightforward. Members were then invited to provide further feedback on Draft 2, with further amendments made where relevant.

### 2.3 Participants

Participants in this study were 13 members of the Reference Group that supported the development of the National Framework. Two had represented community organisations (including advocates for individuals and professionals), 10 had represented professional organisations (including peak membership bodies for medical and allied health professions), and one had represented a government agency. To be eligible to participate, Reference Group members must have attended at least one meeting and/or provided feedback on at least one draft document. For this co‐production evaluation study, participants contributed in their own personal capacity, based on their experiences, not as representatives of their organisations.

### 2.4 Recruitment

All eligible participants (*n* = 31) were contacted via an initial email outlining the proposed research study in November 2024. Three were uncontactable due to leaving their roles within the organisation or Government Department they were affiliated with during the co‐production process, resulting in 28 individuals receiving the initial correspondence. Eligible participants were given 7 days to respond and if any participants objected, the survey would have proceeded as an internal quality evaluation only; no objections were received. The invitation also provided participants an opportunity to state they did not support the study results being shared in open forums such as journal articles and conference presentations.

A follow‐up email was sent after 7 days to invite participants to complete an online, anonymous survey evaluating their perspectives on participating in Reference Group activities and overall co‐production process. At this stage, participants were invited to express their interest via email in contributing to any research outputs as a co‐author, and were also asked ‘*Would you like to contribute to research outputs (e.g., journal article, conference presentation) as a co‐author?*’ at the beginning of the online survey. This option provided an opportunity for true co‐production where all group members were able to actively contribute to the study.

### 2.5 Survey

The online survey developed for the current study was informed by a measure designed by Coulston, Spittle [14] as part of the development and evaluation of their Partnership‐focussed Principles‐driven Online co‐Design (P‐POD) Framework. The P‐POD Framework focuses on four areas: *inclusive* (valuing difference), *respectful* (equitable partnerships), *goal directed* (person and process) and *supporting participation*. These were expanded upon (e.g., we were interested in specifically evaluating accessibility and helpfulness of strategies employed) and evaluated in the survey via a combination of closed and open questions. The survey asked participants to reflect on the extent to which the co‐production process was *accessible, inclusive, respectful, participatory, outcomes focussed*, and *helpful*. For most closed questions, participants were asked to rate their level of agreement on pre‐generated statements using a 5‐point Likert response scale (*strongly disagree, disagree, neutral, agree, strongly agree*). For statements related to how *helpful* each aspect of the co‐production process was, participants rated their level of agreement using a 3‐point Likert response scale (*very helpful, neither helpful nor unhelpful, unhelpful*). Additional open response items were included to allow participants to elaborate on their responses and/or provide alternative responses that were not included in the pre‐generated statements. The survey was delivered online using Qualtrics and took ~10–15 min to complete. A copy of the survey is available on request.

### 2.6 Data Analysis

All data were downloaded from Qualtrics and imported into SPSS version 29. Descriptive analysis of quantitative data was conducted to obtain frequencies and percentages of responses per survey item. Responses from open‐ended questions are used for illustrative purposes and presented verbatim. Participant ID numbers are used to help illustrate the distribution of quotes across participants. Results are presented according to the constructs examined in the survey and then synthesised in the discussion using recommendations by Nordin, Kjellstrom [10] that evaluation focus on co‐production *outputs, experience*, and *outcomes*.

### 2.7 Manuscript Co‐Production

Participants expressing interest in contributing to research outputs were contacted and the manuscript was then co‐produced through a series of online meetings and iterative reviews of shared draft documents. Members of the Reference Group were invited to contribute in ways that aligned with their preferred mode of engagement including drafting sections, providing verbal and/or written feedback and contributing to critical review of draft versions. The final manuscript was shared and agreement sought from all co‐authors prior to submission for publication.

### 2.8 Positionality

Six authors (EB, RW, ZYN, LG, KE, and DT) were members of the project team and five were members of the Reference Group involved in co‐production (BC, AC, CG, AL, and EW). Three authors publicly identify as neurodivergent and/or having disability (RW, BC, and CB), and all authors have allied health qualifications, allied health research experience, and/or are practicing clinicians.

## 3 Results

All 13 participants who consented to participate completed the survey. Findings are organised according to the constructs evaluated in the survey. A synthesis of findings regarding participants' reflections on the *approach, experience*, and *outcomes* is provided in the discussion. Table 1 presents the frequencies and percentages of responses for statements related to the accessibility, inclusiveness, respectfulness, and outcomes of the co‐production process. Across all 28 statements and 356 responses, the majority (94%; *n* = 335) were positive *(agree, strongly agree*). Four percent of responses (*n* = 14) were neutral, and 2% (*n* = 7) negative (*disagree* or *strongly disagree*).

**Table 1:** Frequencies and percentages of responses for statements related to the accessibility, inclusiveness, respectfulness, participatory, and outcomes of the co‐production process.

Survey section	Statement	Strongly disagree	Disagree	Neutral	Agree	Strongly agree
Accessible	It was easy to access documents related to RG meetings.				4 (30.8%)	9 (69.2%)
	It was easy to use the Microsoft Teams platform for meetings.		1 (7.7%)		4 (28.6%)	8 (61.5%)
	It was easy to contribute to RG meetings.				6 (46.2%)	7 (53.8%)
	It was easy to contact members of the project team.			1 (7.7%)	1 (7.7%)	11 (84.6%)
	It was easy to participate in the project in a way that suited my needs and/or preferences.				4 (30.8%)	9 (69.2%)
Inclusive	I felt included in the meetings.				3 (23.1%)	9 (76.9%)
	I felt I had enough support to contribute if/when I wanted to.				1 (7.7%)	12 (92.3%)
	I felt that my contributions were valued.				3 (23.1%)	9 (76.9%)
	I felt that my contributions were utilised.			1 (7.7%)	2 (15.4%)	9 (76.9%)
	The RG brought together diverse stakeholder groups.				2 (15.4%)	11 (84.6%)
	The RG made space for and supported non‐judgemental debate.				3 (23.1%)	10 (76.9%)
Respectful	I felt respected by other RG members.				2 (15.4%)	11 (84.6%)
	I felt respected by the project team.				1 (7.7%)	12 (92.3%)
	I felt like I was seen as an expert representing my organisation.				2 (15.4%)	11 (84.6%)
	I felt like I was seen as an expert in my own lived experience.^a^			1 (8%)	1 (8%)	6 (50%)
	I felt listened to.			1 (7.7%)	1 (7.7%)	11 (84.6%)
	I felt that my input had equal standing to other people's input.			2 (15.4%)	3 (23.1%)	8 (61.5%)
	I felt strategies were implemented to ensure people were respected.				4 (30.8%)	9 (69.2%)
	I felt the project team was transparent about power and aimed to recognise and reduce the effects of power imbalances.				5 (38.5%)	8 (61.5%)
	I felt the project team facilitated relationship‐building between the group.				5 (38.5%)	8 (61.5%)
Participatory	I felt like I could participate if/when I wanted to.			1 (7.7%)	2 (15.4%)	10 (76.9%)
	I felt like meetings were open and responsive.				3 (23.1%)	10 (76.9%)
	I felt that feelings and experiences were shared in meetings without judgement.				4 (30.8%)	9 (69.2%)
Outcomes‐focused	I felt like the RG activities (e.g. meetings, review of documents) worked towards clear aims.	1 (7.7%)			6 (46.2%)	6 (46.2%)
	The process was open to new ideas, or to refining ideas and solutions.	1 (7.7%)		2 (15.4%)	4 (30.8%)	6 (46.2%)
	I felt like the RG activities achieved those aims.	1 (7.7%)		1 (7.7%)	6 (46.2%)	5 (38.5%)
	I felt like the Framework represents my contributions in the RG activities and draft documents.	1 (7.7%)	1 (7.7%)	1 (7.7%)	6 (46.2%)	4 (30.8%)
	I felt proud of the final Framework document.	1 (7.7%)		3 (23.1%)	4 (30.8%)	5 (38.5%)

^a^ Participants had the option to not provide a response if this item was not applicable to them (*n* = 8).

### 3.1 Accessible

All Reference Group members indicated it was *easy to* contribute to Reference Group meetings, *participate in the project* in a way that suited their needs and/or preferences, and *access meeting‐related documents*. Participant 2 noted ‘I appreciated the team sending me power points prior to the meeting so that I was able to access them more easily to suit my vision issues.’ Participant 6 explained ‘It was wonderful to have any accessibility needs provided for as a matter of course, without having to ask, or any judgement if you used them.’ Most participants also indicated they found it *easy to use the Microsoft Teams platform* for meetings and to *contact members of the project team*. One participant reported experiencing challenges using Microsoft Teams, while another responded *neutral* to the statement: *It was easy to contact members of the project team*.

### 3.2 Inclusive

As evidenced in Table 1, all participants indicated they felt *included in the meetings* and *had enough support to contribute if/when they wanted to*. Participant 13 highlighted the importance of the team working deliberately to create and foster an inclusive work environment:

I found the reference group to be a highly inclusive environment, where contributions were actively encouraged via a range of channels (by speaking up during the meeting, typing into the chat and via follow up communication with the project team outside the meeting), ensuring options for all styles of communication and accessibility. I was impressed by the project team's deliberate efforts to foster an inclusive environment. They demonstrated that inclusivity is central to their process by addressing it overtly and actively seeking feedback on their approach to inclusivity at the start of each meeting.

There was also broad agreement that the Reference Group *brought together diverse stakeholder groups* and *made space for and supported non‐judgemental debate*. Participant 4 explained how elements of the meeting structure helped foster inclusion:

A wonderful group where diversity was celebrated, and inclusive practices were utilised. I particularly appreciated the opportunity for providing feedback at the end of the meetings and that any feedback given was incorporated into future meetings (e.g., around topics like accessibility). This rolling agenda item made it feel safe to give feedback and positively role‐modelled that feedback was welcome/would be taken on (not that I needed to but had I it would have been easy to do so).

All but one Reference Group member indicated they *felt that their contributions were utilised towards the co‐production outcomes*, with this individual remaining *neutral*. Qualitative information to contextualise the neutral response was not provided.

### 3.3 Respectful

All participants reported feeling *respected by the project team* and *other Reference Group members*, *seen as an expert representing their organisation*, and agreed *strategies were implemented to ensure people were respected*. Participant 6 shared it was ‘honestly the most respectful process I have EVER been a part of in almost 18 years of practice. It set the tone for everyone's behaviour, and it meant if someone wasn't being respectful (only noted once) it really stood out.’ Participant 2 commented on the importance of reciprocity in the co‐production process, noting that ‘Getting personal responses and thanks for contributing was lovely.’

The project team was perceived as *being transparent about power*, aimed to *recognise and reduce the effects of power imbalances*, and *facilitated relationship‐building between members of the group*. Participant 13 commented on the importance of creating a safe space for different perspectives to be shared:

This was a highly respectful process. I felt that all participants' viewpoints and priorities were valued by the project team and fellow reference group members. The facilitators were intentional with creating a safe space for open dialogue, where differing perspectives were welcomed and thoughtfully considered.

However, it should be noted two participants reported *neutral* ratings in terms of *feeling that they were listened to, that their input had equal standing to that of other people in the group, and/or were seen as an expert in their own lived experience*. Qualitative data to contextualise these ratings were not provided.

### 3.4 Participatory

There was consensus amongst participants that *meetings were open and responsive*, and the *feelings and experiences of members were shared in meetings without judgement*. All but one Reference Group member, who responded *neutral*, reported they felt they *could participate in the co‐production activities if/when they wanted to*. Participant 2 commented ‘It was very interactive, and I could see that my feedback was incorporated and balanced with other perspectives.’ Participant 4 explained ‘It was such a positive and seamless process, one I have never experienced before!’

However, there were differing views regarding the meeting structure and the extent this fostered participation. For example, Participant 13 noted that how sessions were organised (twice monthly meetings with a predictable co‐production cycle) contributed to a positive experience:

I found the structure and pace of the reference group sessions to be respectful of the co‐production process ‐ allowing time for me to understand the context, key concepts, develop and share my own ideas and reflect on the contributions of others. The process did not feel rushed, despite the enormity of the project. This method of co‐production was successful in managing the diverse contributions of quite a large reference group. It allowed for continued iteration and refinement of ideas and content throughout the process, as our shared understanding of the scope of the framework developed over time.

However, Participant 6 expressed mixed feelings about their participation:

It was great, particularly the cycles approach whereby you could see feedback shaping the final product in an ongoing way. It is a big‐time commitment though, especially at lunch time on a Friday, I would probably prefer a different day/time in future.

Similarly, Participant 7 acknowledged positive aspects of the meetings, but indicated they would have preferred greater involvement beyond providing feedback on the draft documents towards the end of the project:

I thought that the coproduction provided the opportunity for gathering perspectives from a community, but don't think it was as successful in bringing together diverse literature and academic strengths of the disciplines… I felt that I was able to make comment and discuss, but I didn't feel that I was part of the final resolution of the document or the work in an academic sense…Given the big time commitment, I'm not sure that my final contribution made the most of this time..

### 3.5 Outcomes‐Focused

Most Reference Group members indicated *agree* or *strongly agree* with statements related to various outcomes of the co‐production process. Over three quarters of participants agreed or strongly agreed the co‐production process was *open to new ideas*, or to *refining ideas and solutions*, and the resulting Framework document *reflected the contributions they had made throughout the process*. For example, Participant 1 explained ‘Co‐production was genuinely evident and practiced.’ Participant 2 commented on the influence they felt their contributions had on the developed of the Framework, noting that ‘I felt like my feedback was incorporated in the drafts.’

One participant chose ‘strongly disagree’ for all statements in the Outcomes section, which was the only section of the survey to receive this rating. However, there is a strong possibility this was done in error, given the participant's pattern of responding across the survey (i.e., all ‘strongly agree’ or ‘very helpful’) and the fact that all of their qualitative responses were also very positive (e.g., ‘This was one of the very best Reference Groups that I have belonged to’). Nevertheless, feedback from Participant 7 revealed concerns regarding the way in which individual contributions were cited in the final document, including the role of representatives in informing, versus authoring, the National Framework:

I think that some of my intellectual property went into the work, but that this hasn't been acknowledged in an academic sense.

### 3.6 Helpful

Participants' responses to questions regarding the helpfulness of various co‐production strategies are presented in Table 2. Across all 8 statements and 104 responses given, the majority (81%; 84) indicated the strategies used were ‘very helpful’. Seventeen percent [15] reported ‘neither helpful nor unhelpful’, and 2% [2] ‘unhelpful’. Elements that were reported as most helpful included the presentation of co‐production points, review of previous co‐production points, small group discussions in break‐out rooms, whole‐group discussions, and review of the draft Framework documents. Participant 12, for example reflected positively on ‘the thorough explanation of the co‐production points’ and Participant 2 commented ‘the breakout rooms allowed for a greater contribution and for all perspectives to be heard.’

**Table 2:** Frequencies and percentages per statement related to the helpfulness of co‐production strategies used with the reference group.

Element	Very helpful	Neither helpful nor unhelpful	Unhelpful
Meeting introduction (Acknowledgement of Country, meeting overview, minutes, updates).	9 (69.2%)	3 (23.1%)	1 (7.7%)
Review of previous Co‐Production Points in meetings.	11 (84.6%)	2 (15.4%)	
Co‐Production Point presentations.	13 (100%)		
Co‐Production Point small break‐out group discussions.	12 (92.3%)	1 (7.7%)	
Co‐Production Point whole‐group discussions.	11 (84.6%)	2 (15.4%)	
Co‐Production Point polls and/or surveys.	8 (61.5%)	4 (30.8%)	1 (7.7%)
Final comments in meetings.	7 (53.8%)	6 (56.2%)	
Review of draft Framework documents.	13 (100%)		

Participant 13 also reflected on the break‐out rooms, indicating they would have preferred even more time be allocated for this way of working:

I found the review of previous co‐production points, including any summaries of drafted content on the previous co‐production point, to be useful before embarking on the next co‐production point. I found the breakout rooms very helpful and a more inclusive environment for myself to make comments in as I personally prefer smaller group discussions over larger groups. It would have been great to have longer in the breakout rooms if possible. The final comments in every meeting always left me feeling positive about progress, the project and my contributions. Time to review the draft documents was really helpful also.

However, Participant 11 shared a different perspective, which may help contextualise ratings of *neutral*, and in two cases *unhelpful*, when it came to how *helpful* the co‐production strategies were perceived to be:

While I feel it was important to have inclusivity, including those with lived experience, I felt that it was so prioritised that it reduced the functionality of the group. I felt we spent a great deal of time ensuring everyone was given time on every point, that I found the progress of the meetings too slow. There needed to be a more firm chairing of the meetings to ensure appropriate pace of progress was happening.

## 4 Discussion

The objective of this study was to evaluate the experiences of those involved in co‐production of the development of the National Framework, to inform our future co‐production activities and in a way that can contribute to improvements in the wider community. Our survey‐based approach examined how *accessible, inclusive, respectful, participatory, outcomes focussed*, and *helpful* the co‐production process was effective in helping participants reflect on their experiences. In doing so, their insights can now be translated into actionable recommendations to support future co‐production processes. To do this, we will discuss the findings in relation to the three perspectives Nordin, Kjellstrom [10] encouraged research to address, when evaluating co‐production research: *outputs*, *experiences*, and *outcomes*.

### 4.1 Co‐Production Outputs

A common challenge in evaluating co‐production research is a lack of specificity in reporting the processes used [10]. To address this, our team employed two specific methods. The first, which is good practice in all research, was to thoroughly document the methods used, including a detailed account of the process that included participants, processes, and outcomes (see Supporting Information; 13). Second, our development and use of the CPP methodology supports specificity and transparency in reporting. This provided an organised, transparent, and systematic way of conducting and documenting the co‐production process, including how the information gathered was used.

Co‐production outputs refer to the results of the co‐production processes [10], including the *approach* employed. Our findings in relation to co‐production outputs, and the processes underpinning them, however, were not uniformly positive. For example, a minority of participants shared neutral, and on two occasions *unhelpful* ratings in relation to the helpfulness of specific co‐production strategies. These ratings related to aspects of how online meetings were conducted, including the use of polls, break‐out rooms, and the sharing of final summary comments by the co‐chairs. The variation in ratings seems to reflect individual preferences. For example, with the *final comments* at the end of each meeting, which focused on acknowledging and thanking members of the Reference Group for their contributions, seven participants rated *very helpful*, and six participants rated *neither helpful nor unhelpful*.

Accommodating individual needs and preferences in group‐based work situations is often challenging, and this co‐production process was no different [16, 17]. For example, we found differing views regarding the nature and pace of co‐production activities. Whereas for one participant, ‘the structure and pace of the reference group sessions to be respectful of the co‐production process ‐ allowing time for me to understand the context, key concepts, develop and share my own ideas and reflect on the contributions of others’ for another, ‘… (inclusivity) was so prioritised that it reduced the functionality of the group.’ These differences in views reinforce the importance of understanding each member's needs and preferences, and establishing expectations, at the outset, and then monitoring and if necessary, re‐calibrating throughout the process.

### 4.2 Co‐Production Experience

In reviewing the participants' responses, particularly the qualitative reflections, it was apparent that the *experience* was of keen interest to most. For example, when asked about accessibility, which on the face of it relates to practical considerations, participants' comments tended to focus on their positive feelings about proactivity within the project team to meet these needs. It was also noteworthy that some comments conveyed a sense of *surprise* at the effective implementation of genuine co‐production practices and the positive experiences this created. This finding suggests at least some participants came to the project with potential low expectations, and even scepticism, of co‐production based on prior experience (e.g., Participant 3, Participant 15). This is a common challenge in co‐production where participants bring differing levels of experiences, priorities and perceptions to the process [15, 18]. As such, it is important that co‐production includes early understanding of roles and responsibilities, expectations and shared‐decision making processes to support meaningful participation and facilitate successful outcomes [16, 18]. Our findings suggest that when inviting participants to co‐produce research, it may be helpful to discuss their prior experiences where they feel comfortable to do so, to support open discussion about what worked well, not well, and how the planned processes will compare. This finding also provides further evidence to support the value of sharing transparent evaluations of co‐production research, which highlight both the strengths and shortcomings to encourage an open conversation about what works for individual participants, and why [19].

### 4.3 Co‐Production Outcomes

The findings point to overall positive perceptions of the outcomes of the project amongst participants. To contextualise these findings, all co‐production activities ran according to schedule. The National Framework was delivered on time and with positive feedback from community, professional, and Government stakeholders. However, the survey results relating to outcomes are in fact the most mixed. For example, although 10 of the 13 participants *agreed* or *strongly agreed* that they *felt like the Framework represents my contributions in the Reference Group activities and draft documents*, there was also one response each for *neutral, disagree*, and *strongly disagree*. Our interpretation is that these latter responses likely reflect the challenge that comes with working towards consensus with a large group (in this case, 23) of representatives from community and professional organisations, with each member bringing different views, experiences, and perspectives to the task. This may include different priorities and potential conscious and unconscious biases, in the context of different stakeholder representation and policy influence [17]. In our view, it also demonstrates the importance of separating the approach, experience, and outcomes when evaluating co‐produced research, because it allows for consideration of all possible permutations. For example, it is possible that a participant may reflect positively on the approach and experience but feels unsatisfied with one or more aspects of the outcome.

This interpretation in no way diminishes the importance of seeking to achieve unanimous success in all aspects of approach, experience, and outcomes. Indeed, our findings reinforce the need to ensure there are robust methods in place to encourage and support participants to share their views, experiences, and any concerns throughout the process. For this project, strategies to support communication and consensus building included regular check‐ins within and outside meetings, an open anonymous online survey (with the features ensuring anonymity openly explained and demonstrated to the group), multiple cycles of review on draft documents, individual meetings with members regarding their feedback, and an ongoing open invitation to meet with the project co‐chairs. However, it is possible challenges arise not so much from openness and opportunities for communication, but rather misalignment regarding expectations. For example, feedback from one participant, indicating they expected their individual contributions would be cited in the document appears to reflect a misalignment of understanding. Instead, the methodology employed focused on synthesising evidence from all sources (research review, environmental scan, community consultation, yarning, and CPPs) and then translating this into plain language, actionable advice. For future, we recommend including an explicit statement regarding how individual contributions will be acknowledged (e.g., acknowledgement of contribution *vs.* individual citations) in the Terms of Reference for the Reference Group and published documents.

### 4.4 Limitations

This study was effective in eliciting diverse and insightful reflections on co‐production, but the findings should be considered in the context of several limitations. First, although the response rate was relatively high at 46% it is just under half of all potential participants. Participants who participated in the Reference Group but did not respond to the survey may have offered different perspectives. Nevertheless, the fact there was diversity in the views expressed and we provided potential participants with the opportunity to indicate that they did not support the results of the study being shared, bolsters confidence in the credibility of the findings. It is also noteworthy that the response items for questions regarding helpfulness of co‐production activities were not completely balanced, meaning it may have led to more neutral or *unhelpful* ratings. Specifically, the only option of participants to express positive sentiment was *very helpful*. This should have been *helpful* which would have balanced *unhelpful* in the opposite direction and presumably had a lower threshold for selection. This may account for the larger number of *neither helpful nor unhelpful* responses to items in this section of the survey.

### 4.5 Future Directions

The findings of this study suggest that some of the methods we developed and used may be helpful to other teams when working with groups that involve clinicians, consumers and researchers as a way to assist in engaging in co‐production of policies, guidelines, frameworks and other documents. From a practical perspective, we found the use of *CPPs* particularly helpful in structuring the process, by enabling us to move systematically through a range of topics and issues, and document outcomes and report findings transparently. Future studies should consider using CPPs to help structure the co‐production process.

From a research perspective, we found recommendations by Nordin, Kjellstrom [10] that evaluation focus on co‐production *outputs, experience*, and *outcomes*, both helpful and feasible in analysing and interpreting results. We note, however, the interconnected nature of these three outcomes (e.g., an inclusive approach resulting in positive experience), which is why we opted for a construct‐based examination in the results, followed by synthesis using this method in the discussion. We encourage future studies to employ the *outputs, experience*, and *outcomes* framework, but also support flexibility in its application based on our experience.

From a personal perspective, as a team, we are on a journey, and our methods will, and must, continue to evolve. Sharing our findings, including the strengths and challenges, is intended to support progress in co‐produced research. We warmly encourage other researchers to continue to similarly share the full richness of experiences, and outcomes, including areas to improve, as we work together to employ, evaluate, and ultimately further enhance co‐production methodologies.

## 5 Conclusion

This study provides a detailed account of the co‐production process undertaken in developing the National Framework for assessing children's functional strengths and support needs and participant reflections that can directly inform future co‐production efforts. The findings demonstrate the importance of considering co‐production outputs, experiences, and outcomes independently, and in an interconnected manner. Doing so enables a nuanced understanding of participant experiences, views, and reflections but in a way that also supports aggregation and comparison of findings across studies. In practical terms, the study points to the importance of well‐organised, respectful, and purposeful engagement in co‐production and provides examples of strategies that can support such an approach.

## Author Contributions

**Emmah Baque:** conceptualization, methodology, formal analysis, writing – original draft, writing – review and editing, data curation. **Rachelle Wicks:** conceptualization, methodology, formal analysis, writing – original draft, writing – review and editing. **Zheng Yen Ng:** conceptualization, methodology, formal analysis, writing – original draft, writing – review and editing. **Libby Groves:** conceptualization, methodology, formal analysis, writing – original draft, writing – review and editing, project administration. **Bianca Comfort:** writing – original draft, writing – review and editing. **Amanda Curran:** writing – original draft, writing – review and editing. **Clare Gibellini:** writing – original draft, writing – review and editing. **Ailsa Leslie:** writing – original draft, writing – review and editing. **Erin West:** writing – original draft, writing – review and editing. **Kiah Evans:** conceptualization, methodology, writing – original draft, writing – review and editing. **David Trembath:** conceptualization, methodology, writing – review and editing, writing – original draft, funding acquisition, formal analysis.

## Ethics Statement

The study received ethical approval from The Griffith University Human Research Ethics Committee (GU Ref No: 2024/814), which included coverage for research participants (i.e., members of the co‐production group who volunteered to engage in this follow‐up study) to both identify themselves and act as co‐authors.

## Conflicts of Interest

The authors declare no conflicts of interest.

## Data Availability

The data that support the findings of this study are available on request from the corresponding author. The data from this study are not publicly available due to ethical and privacy restrictions.

## Appendix 1

**Example of Co‐Production Point**

**Co‐Production Point:** 2

**Related Meeting/s:** 1 March (Meeting 3)

**Framework section:** Guiding Principles

**Lead/s:** Rachelle Wicks and Libby Groves

**What are we seeking?**

We would like to hear your views about guiding principles that should underpin the Framework. We have a wide range of organisations in the Reference Group, and would like to explore what principles are particularly relevant to your organisations and the communities you represent and support.

**Why is it needed?**

Guiding principles will provide a foundation for the Framework, and work across all aspects including assessment, differentiation, and reporting. Gathering information about Principles used across a range of professions and sectors will also ensure that the Framework is relevant more broadly and encompasses the intended scope.

**What do we know?**

The Project Team is completing a systematic research review and search of the web (grey literature search) to identify relevant Principles from existing frameworks and policy documents within the child health, education, and wellbeing space. Work to date has shown that many frameworks share Principles founded upon on United Nations Convention on the Rights of the Child (UNCRC) and person‐centred practice, as well as Principles that are more specific to a particular framework/sector.

**What don't we know?**

We know that the review will not capture all relevant Principles across professional groups and other organisations within the child health, education, and wellbeing space, and that you as RG members are ideally placed to share suggestions about what other information may be helpful, from a range of perspectives.

**Co‐Production Activity**

- ***Before the meeting***
  As a representative of your organisation, please reflect on what guiding principles you believe should be the foundation for the Framework. If possible, write these down for discussion during the meeting. We are keen to know what the principles are, and why they are important.
- ***During the meeting***
  We will break into small groups and ask members to share suggestions via talking or text functions. We'd encourage you to share 2‐3 Principles that are particularly important for guiding best policy and/or practice within your organisation and discuss why they are important. If you have any additional points that you do not get to discuss, we encourage you to add these to the chat.
- ***After the meeting***

We will compile the information you share, and then combine it with information from the research review, grey literature search, and community consultation to identify a small number of guiding principles. We will share a draft with you for further discussion.

**Additional Information**

We anticipate identifying many potential principles, but will be seeking to distil these down to just a small number (e.g., 3 to 5). However, for this exercise, please share what is most important to your organisation. The process of distiling can happen afterwards. In the groups, we will ask you to share 2‐3 of your top priorities, but you are most welcome to share a longer list via chat during the meeting or email.
